# Draft Genome Sequence of the Plant Growth Promoter and Biocontrol Agent Bacillus velezensis Strain BIB0110

**DOI:** 10.1128/mra.00231-23

**Published:** 2023-05-31

**Authors:** Isabela de Carvalho, Gladys Angélica Apaza-Castillo, Isabel Cristina Padula Paz, Alexandre Martins Guimarães, Maria Carolina Quecine, Maria L. Bonatelli

**Affiliations:** a Department of Genetics, Luiz de Queiroz College of Agriculture, University of São Paulo, Piracicaba, Brazil; b Biota Innovations, Uberaba, Brazil; c Department of Environmental Microbiology, Helmholtz Centre for Environmental Research—UFZ, Leipzig, Germany; University of Arizona

## Abstract

Here, we report the draft genome sequence of Bacillus velezensis strain BIB0110, a broad-range biocontrol agent isolated from cultivated eucalyptus in Brazil. The genome has a size of 4.19 Mbp, with a GC content of 45.87%, and it was assembled into 32 scaffolds.

## ANNOUNCEMENT

Plant growth-promoting bacteria (PGPB) are bacteria that upon establishment on a plant or in its surroundings, can act as a biofertilizer or biocontrol agent. This is an important association, since it can result in the reduction of fertilizers and pesticide in agriculture, while maintaining crop productivity and safety (1, 2).

Bacillus velezensis strain BIB0110 is an endophytic bacterium previously isolated from cultivated eucalyptus in Brazil (3). *In vitro* assays showed the potential of this strain to solubilize phosphate, fix atmospheric nitrogen, and produce indoleacetic acid (3). Eucalyptus plantlets inoculated with BIB0110 showed a significant increase in the growth of root and aerial parts (3). More interestingly, BIB0110 significantly inhibited the mycelial growth of the fungal pathogens Botrytis cinerea and Calonectria gracilis, and it also reduced the incidence and severity of the disease incited by these pathogens on eucalyptus plants (4). The authors reported that the inhibition potential may have been related to diffusible and/or volatile metabolites (4).

Here, we report the draft genome sequence of *B. velezensis* strain BIB0110 and the portfolio of genes involved in its potential to act as a PGPB, in particular, as a biocontrol agent.

*B. velezensis* strain BIB0110, previously named *B. amyloliquefaciens* EUCB10 (3, 4), was isolated from Eucalyptus urophylla × Eucalyptus grandis hybrids cultivated in Brazil (3). A single colony was picked and grown in liquid tryptone soya broth (Oxoid, UK) at 28°C under agitation for 20 h. Genomic DNA was extracted using the DNeasy blood and tissue kit (Qiagen, Germany) according to the manufacturer’s instructions. A library was prepared with genomic DNA (1 ng) using the Nextera XT DNA library kit (Illumina, USA). The whole genome was sequenced on the Illumina MiSeq platform using the v. 3 reagent kit (Illumina) to yield 2 × 250-bp paired-end reads. Sequencing was performed at NGS Genomic Solutions (Piracicaba, Brazil).

The raw sequences (total 3.92 million reads, corresponding to 238× genome coverage) were trimmed and merged using HTStream v. 1.3.3 (https://github.com/s4hts/HTStream). SPAdes v. 3.14.1 (5) was used to assemble the contigs considering the flags –isolates and –cov-cutoff “auto,” while Platanus v. 1.2.1 (6) was used to scaffold the genome. All software was used with default parameters, unless otherwise specified.

The genome of *B. velezensis* strain BIB0110 has a size of 4,192,465 bp and a GC content of 45.87%, and it was assembled into 32 scaffolds with an *N*_50_ value of 642,639. The completeness and contamination, estimated using CheckM (7), were 99.81% and 0%, respectively. Annotation was performed using the NCBI Prokaryotic Genome Annotation Pipeline (PGAP) and revealed 4,260 genes, with 4,155 coding sequences and 105 noncoding sequences (17 rRNAs, 83 tRNAs, and 5 noncoding RNAs [ncRNAs]).

AntiSMASH v. 6.1.1 was used to identify secondary metabolites biosynthesis gene clusters, setting the detection strictness as relaxed (8). Interestingly, we predicted genes encoding secondary metabolites with known antibiotic activity, such as bacilysin (9), difficidin (10), bacillaene (11), macrolactin H (12), surfactin (13), and fengycin (14) (Table 1). This indicates that BIB0110 harbors important genes to promote the biocontrol of pathogens with more sustainable approaches.

**TABLE 1:** Secondary metabolite clusters with similarity values of >95% identified in Bacillus velezensis BIB0110 using antiSMASH 6.1.1^*a*^

Cluster	Type	Contig	Position (bp)	Most similar cluster	MIBiG	Similarity (%)
1	Other	JAOWMS010000001	313118–354536	Bacilysin	BGC0001184	100
2	RiPP-like, NRPS	JAOWMS010000001	876727–928516	Bacillibactin	BGC0000309	100
3	transAT-PKS	JAOWMS010000002	98127–191924	Difficidin	BGC0000176	100
4	transAT-PKS, NRPS, T3PKS	JAOWMS010000003	60781–161427	Bacillaene	BGC0001089	100
5	transAT-PKS	JAOWMS010000003	424081–512314	Macrolactin H	BGC0000181	100
6	NRPS	JAOWMS010000004	210792–276199	Surfactin	BGC0000433	95
7	NRPS, betalactone, transAT-PKS	JAOWMS010000006	2833–140622	Fengycin	BGC0001095	100

a RiPP, ribosomally synthesized and posttranslationally modified peptide; NRPS, nonribosomal peptide synthase; transAT-PKS, *trans*-acyltransferase polyketide synthase; T3PKS, type III polyketide synthase; MiBIG, Minimum Information about a Biosynthetic Gene cluster.

### Data availability.

This whole-genome shotgun sequencing project has been deposited at DDBJ/ENA/GenBank under the accession no. JAOWMS000000000. The version described in this paper is the first version, JAOWMS010000000, and consists of sequences JAOWMS010000001 to JAOWMS010000032. The raw sequence reads were deposited in the Sequence Read Archive under the accession no. SRS17593923.
